# Dissection of Targeting Molecular Mechanisms of Celastrol-induced Nephrotoxicity *via* A Combined Deconvolution Strategy of Chemoproteomics and Metabolomics

**DOI:** 10.7150/ijbs.91751

**Published:** 2024-08-26

**Authors:** Xueying Liu, Qian Zhang, Peili Wang, Xin Peng, Yehai An, Junhui Chen, Jingnan Huang, Shuanglin Qin, Hengkai He, Mingjing Hao, Jiahang Tian, Letai Yi, Ming Lei, Piao Luo, Jigang Wang, Xinzhou Zhang

**Affiliations:** 1 Department of Nephrology, Guangdong Provincial Clinical Research Center for Geriatrics, Shenzhen Clinical Research Center for Geriatric, Shenzhen People's Hospital, The First Affiliated Hospital, School of Medicine, Southern University of Science and Technology, Shenzhen, Guangdong 518020, China.; 2 School of Traditional Chinese Medicine and School of Pharmaceutical Sciences, Southern Medical University, Guangzhou 510515, China.; 3 Shenzhen Key Laboratory of Kidney Diseases, Shenzhen People's Hospital (The Second Clinical Medical College, Jinan University; The First Affiliated Hospital, Southern University of Science and Technology), Shenzhen 518020, China.; 4 Inner Mongolia Medical University, Hohhot, 010107, China.; 5 State Key Laboratory for Quality Ensurance and Sustainable Use of Dao-di Herbs, Artemisinin Research Center, and Institute of Chinese Materia Medica, China Academy of Chinese Medical Sciences, Beijing 100700, China.; 6 Ningbo Municipal Hospital of TCM, Affiliated Hospital of Zhejiang Chinese Medical University, Ningbo 310060, China.; 7 National Clinical Research Center for Chinese Medicine Cardiology, Xiyuan Hospital, China Academy of Chinese Medical Sciences, Beijing 100091, China.; 8 National Engineering Research Center of Personalized Diagnostic and Therapeutic Technology, TCM Precision Medicine Research Department, FuRong Laboratory, Hunan University of Chinese Medicine, Changsha, Hunan 410007, China.; 9 Department of Nephrology, Guangzhou Eighth People's Hospital, Guangzhou, Guangdong 510440, China.

**Keywords:** celastrol, nephrotoxicity, chemical proteomics, metabolomics, mitochondrial dysfunction, apoptosis

## Abstract

Celastrol (Cel), derived from the traditional herb *Tripterygium wil*fordii Hook. f., has anti-inflammatory, anti-tumor, and immunoregulatory activities. Renal dysfunction, including acute renal failure, has been reported in patients following the administration of Cel-relative medications. However, the functional mechanism of nephrotoxicity caused by Cel is unknown. This study featured combined use of activity-based protein profiling and metabolomics analysis to distinguish the targets of the nephrotoxic effects of Cel. Results suggest that Cel may bind directly to several critical enzymes participating in metabolism and mitochondrial functions. These enzymes include voltage-dependent anion-selective channel protein 1 (essential for maintaining mitochondrial configurational and functional stability), pyruvate carboxylase (involved in sugar isomerization and the tricarboxylic acid cycle), fatty acid synthase (related to β-oxidation of fatty acids), and pyruvate kinase M2 (associated with aerobic respiration). Proteomics and metabolomics analysis confirmed that Cel-targeted proteins disrupt some metabolic biosynthetic processes and promote mitochondrial dysfunction. Ultimately, Cel aggravated kidney cell apoptosis. These cumulative results deliver an insight into the potential mechanisms of Cel-caused nephrotoxicity. They may also facilitate development of antagonistic drugs to mitigate the harmful effects of Cel on the kidneys and improve its clinical applications.

## Introduction

Celastrol (Cel), a natural pentacyclic triterpenoid product with definitive and divergent therapeutic effects, is among the most promising modern drug candidates^1^. First isolated from the traditional Chinese herb *Tripterygium wilf*ordii Hook. f., it has been long used to treat rheumatoid arthritis^2^, ^3^, and was identified as a main constituent exerting anti-inflammatory^4^ and immunoregulatory functions^5^. Subsequent studies revealed other pharmacological activities of Cel, including anti-tumor^6^, anti-obesogenic^7^, and neuroprotective effects^8^, with growing interest in its potential as a therapeutic agent. However, one obvious limitation of Cel-related clinical applications is its severe side effects, which include reproductive toxicity^9^, hepatotoxicity^10^, and cardiotoxicity^11^. Illustrating the molecular mechanisms of the toxic effects of Cel would facilitate developing strategies for improving its efficacy and reducing its side effects. Although renal tubular injury and necrosis of renal tubular epithelial cells have been associated with Cel, the mechanisms of nephrotoxicity are poorly understood.

Mitochondria have critical effects on homeostatic regulation, including substance metabolism, apoptosis, and autophagy^12^. And mitochondrial damage is an important factor in drug toxicity^13^. Some natural products induce mitochondrial-related cellular apoptosis, contributing to mitochondrial dysfunction^14^, ^15^. In addition, excessive accumulation of aristolochic acid in mitochondria-rich proximal tubular epithelial cells evokes apoptosis, eventually leading to nephrotoxicity^15^, ^16^. Based on our previous confirmation that Cel liposomes can selectively target proteins in the mitochondrial outer membrane^17^, we hypothesized that Cel-induced nephrotoxicity is associated with mitochondrial function.

In recent decades, the development and application of chemical proteomics have provided researchers with new perspectives for studying the molecular mechanisms of drug effects^18^, ^19^. In particular, activity-based protein profiling (ABPP) is effective for providing a comprehensive understanding of the pharmacological activity, and toxic mechanisms of target compounds, through a combination of activity-based probes and proteomics technologies^19^-^21^. We previously used this approach to identify the targets of action of various natural products. For example, aristolochic acid directly affects several crucial enzymes involved in energy metabolism, eventually causing kidney dysfunction^15^, while capsaicin exerts its therapeutic effect on sepsis by inhibiting pyruvate kinase 2 (PKM2)-LDHA-dependent aerobic glycolysis through direct binding to the cysteine residue of PKM2^22^.

Herein, following our previous chemosynthetic strategy, Cel-probe (Cel-p) was designed with a clickable alkyne tag, based on Cel structure^23^, ^24^. This localized structural change has a negligible effect on Cel pharmacological activity^20^. In addition, covalent binding between Cel-p to fluorescent tags (TAMRA-azide) or affinity tags (biotin-azide) *via* a click reaction enables visualization of Cel-targeted proteins, and subsequent enrichment and identification^20^, ^23^. Using chemical proteomics based on ABPP and metabolomics, we analyzed and identified the covalent binding targets of Cel in the kidney.

Our findings indicated that numerous Cel-targeted proteins participate in several vital biological activities, particularly mitochondrial function. Validation of several important enzymes was then undertaken using cellular thermal shift assay-western blotting (CETSA-WB) and pull down-western blotting (PD-WB). Evidence of Cel involvement in multiple metabolic activities and mitochondrial functional impairment was further supported by metabolomics and proteomics analysis, and by measuring JC-1 monomer levels. Our cumulative results imply that Cel nephrotoxicity may function through intricate mechanisms that target specific proteins. Our research provides a prospective approach and new insights into the toxicologic molecular mechanisms of compounds.

## 2. Materials and methods

### 2.1 Reagents

Cel (purity ≥98%) was obtained from Bethealth People Biomedical Technology (Beijing, China). Cel-p, with a clickable alkyne tag, was synthesized and validated as previously described^23^-^25^. Dimethyl sulfoxide (DMSO) was bought from AbMole BioScience (Houston, TX, USA). Cell counting Kit-8 (CCK8) was purchased from Solarbio Life Sciences (Beijing, China). N-Acetyl-L-cysteine (NAC) was sourced from Yeasen Corporation (Shanghai, China). Essential reagents for the click reaction (Tris [3-hydroxypropyltriazolylmethyl] amine [THPTA], vitamin C sodium [NaVc], CuSO4, and TAMRA-azide or Biotin-azide), and liquid chromatography-tandem mass spectrometry (LC-MS/MS) were used, as previously reported^15^, ^20^, ^25^. Of note, in the PD tandem mass tags (TMT) and PD-WB experiment, biotin-azide was added for the click reaction, rather than the TAMRA-azide used in cellular imaging or fluorescence labeling assay *in situ*^20^, ^25^. Sodium deoxycholate (Thermo Fisher Scientific, USA), dithiothreitol (Sigma-Aldrich, Germany), and iodoacetamide (IAA, Sigma-Aldrich, Germany) were used for proteomics analysis^26^. Primary antibodies against Bax, Bcl2, cytochrome C (Cyt C), caspase-3, β-actin, PKM2, and pyruvate carboxylase (PC) were purchased from Proteintech (Chicago, IL, USA). Primary antibodies against fatty acid synthase (FASN) were sourced from Abcam (Cambridge, UK). Primary antibody against voltage-dependent anion-selective channel protein 1 (VDAC1) was obtained from Cell Signaling Technology (Danvers, MA, USA).

### 2.2 Cell culture and viability detection assay

A human renal tubular epithelial cell line (HK-2) was purchased from American Type Culture Collection (Manassas, VA, USA) and cultured as previously reported^15^. A specific density of HK-2 cells was seeded and cultured, in 96-well plates overnight. Subsequently, cells were treated with Cel or Cel-p at different concentrations for 24 h. Cell activity was assessed using the CCK8 kit to determine the effects of treatment of Cel or Cel-p. Densitometric conditions for cell passaging and drug management were as previously described^15^.

### 2.3 Animal experiments

Animal experiment protocols were approved by the Laboratory Animal Care and Use Center of Shenzhen People's Hospital (Shenzhen China, IACUC NO. AUP-230105-LP-569-01). Male (25 ± 5 g, 12 weeks) and female (20 ± 2 g, 12 weeks) C57 BL/6 wild-type mice were housed under standard conditions. Mice were allowed to acclimatize to their environment for 1 week before experimentation. Cel was dissolved in DMSO and diluted with corn oil. Mice in the control group were treated with corn oil. Mice in the treatment group were administered Cel (5 mg/kg/d, intraperitoneally [i.p.]) for 5 days^27^, ^28^. After anesthesia and euthanasia, blood and kidney tissue samples were extracted. Kidney tissues were fixed with paraformaldehyde (PFA) or stored at -80 °C.

### 2.4 Blood biochemistry and histology

Serum levels of creatinine (Crea) and blood urea nitrogen (BUN) were measured by the respective test kits (Jiancheng Biotechnology, Nanjing, China)^29^. After embedding kidney tissues were sliced into 4 μm sections and staining (hematoxylin and eosin [H&E], Masson's trichrome) was carried out to assess histologic changes and evaluate the extent of fibrotic lesions. Kidney tubular injury score was assigned, as described previously^30^.

### 2.5 Mitochondrial function and cell apoptosis detection assay

Cell mitochondrial membrane potential (MMP), detected with the MitoScreen™ (JC-1 staining) kit (Beyotime, Shanghai, China) was assessed according to the proportion of mitochondrial monomers. The CCCP group was used as a positive control for inducing a MMP decrease. BD Pharmingen™ FITC Annexin V Apoptosis Detection Kit (BD Biosciences, USA) was used to detect the percentage of apoptotic cells with Cel treatment^31^.

Adenosine triphosphate (ATP) Assay Kit (S0026, Beyotime, Shanghai, China) was used to detect cellular ATP contents. Cells were washed with cold phosphate-buffered saline (PBS) and then ATP lysate was added, and the supernatant was centrifuged for the ATP test (luminometer, Spark Multifunctional Microplate Tester, Tecan, Switzerland)^32^. NAC pretreatment for 2 h was followed by co-treatment with Cel for 24 h.

### 2.6 Western blotting

Proteins from kidney tissue and cells were detected using polyvinylidene difluoride membranes after being separated on sodium dodecyl sulfate (SDS)-polyacrylamide gel electrophoresis (PAGE). Corresponding primary and secondary antibodies were added to samples followed by incubation. ChemiScope 6100 Fully Automatic Chemiluminescence Imaging System (Qinxiang, Shanghai, China) was used to visualize protein bands using the Omni-ECL™ Femto Light Chemiluminescence Kit (Epizyme, Shanghai, China). Protein expression was semi-quantified following the normalization of WB bands with ImageJ (United States National Institutes of Health, USA).

Based on its simple, reliable principle of thermodynamic stabilization and operational workflow^33^, CETSA was used to measure bond stabilities between Cel and targeted proteins. Briefly, the same quantity of crude protein samples, isolated from HK-2 cells or kidney tissue, were heated with the CETSA heat-pulse machine (Applied Biosystems, Thermo Fisher Scientific, USA) for 5 min after incubation with Cel (20 µM) or DMSO lasting about 30 min^25^. After centrifugation, the supernatant was used for WB assay. The gray value at 37°C was used as a reference for each group, and plotted for analysis^25^, ^34^.

WB experiments on mitochondrial extraction of kidney tissues were conducted using the Tissue Mitochondria Isolation Kit (Beyotime, Shanghai, China)^35^. Briefly, tissue samples were minced, mitochondrial isolation reagent added, supernatant centrifuged to obtain the crude cytosol protein, and precipitate added to the mitochondrial lysate to obtain the mitochondrial crude protein. Next, WB procedures were carried out.

### 2.7 Cellular imaging *in situ*

Fluorescence imaging was implemented, as previously described^34^. A specific number of cells was seeded on four-chamber glass-bottom dishes overnight, after which they were exposed to complete medium containing DMSO or Cel-p (1 µM). Cells were washed after 4 h, then fixed with 4% PFA at room temperature (RT) for 15 min. Samples were washed gently twice and then permeabilized using 0.2% Triton X-100. Cells were pretreated with fresh cocktails (THPTA, NaVc, CuSO4, and TAMRA-azide) prepared according to an established protocol^8^. Then, a click reaction was carried out (2 h, at RT). Cells were cultivated with specific primary antibodies (concentration per manufacturer instructions) overnight at 4 °C. After two rounds of gentle washing with PBS, cells were incubated for 1.5 h with a secondary fluorescent antibody (Alexa Fluor 488; Abcam) at a dilution of 1:1000. Then, cells were washed three times with PBS. Finally, cells were incubated with Hoechst or DAPI for 60 min at RT before image capture, following previously reported procedures^24^.

### 2.8 Fluorescence labeling assay for cells *in situ* and recombinant protein VDAC1

This assay was conducted on cells cultivated at an appropriate density for adaptation. Cells were pretreated with Cel (5 μM, as a competitor) for 1 h (only in the competitor group) and then treated with Cel-p (0.5, 1, 2, or 4 μM) or DMSO for 3-4 h. Proteins were quantified and their same quantities were taken from each group for click reaction with fresh cocktail (THPTA, NaVc, CuSO4, and TAMRA-azide), lasting for 2 h at RT. Precipitation, denaturation, electrophoresis, and visualization of labeled proteins (using fluorescence imaging scanner [Azure C400, USA]) were implemented as described previously^8^, ^24^, ^25^. A similar approach was taken to label and visualize the recombinant protein VDAC1^24^.

### 2.9 Appraisal of pulldown and LC-MS/MS-based targets

Based on the competitive ABPP approach, potent and selective drug targets can be efficiently detected and identified by combined LC-MS/MS analysis^21^. Cells in the competitor group were co-processed with Cel-p (1 μM) for 3 h after Cel pretreatment for 1 h. Subsequently, the extracted protein was subjected to click chemistry for 4 h with fresh click cocktails (THPTA, NaVc, CuSO4, and Biotin-azide)^8^, ^15^, ^25^. Cel-p was bound to streptavidin beads through affinity labeling^25^. Bead-captured targets during the pulldown process were tracked and identified using isotope labeling. A high concentration of SDS (1.5%) was applied to dissolve the protein precipitates obtained from acetone, and stored at low temperatures. Subsequently, protein samples were diluted and centrifuged. They were then co-incubated with beads for 6 h. Peptides were obtained using elution, reduction, alkylation, and enzyme digestion; they were then desalted. Qualitative and quantitative analyses of peptide samples labeled with TMT 10-plex^TM^ Mass Tag Reagent (Thermo, Waltham, MA, USA). Then, labeled peptides were classified and validated with LC-MS/MS (Orbitrap Fusion Lumos, Thermo, USA) following an established protocol^15^, ^24^, ^25^. A sample of the collected protein was set aside for detection by PD-WB before the enzyme reaction^15^.

### 2.10 Label-free proteomics analysis by LC-MS/MS

Proteomics provides insights into potential targets of functional and molecular mechanisms of treatments by analyzing differentially expressed proteins (DEPs) in treated and untreated samples^36^, ^37^. HK-2 cells with Cel or DMSO treatment were lysed in sodium deoxycholate buffer. Then, reduction, alkylation, precipitation, and digestion of lysates in dithiothreitol, IAA, acetone, trypsin, and other solutions were conducted step-by-step. Ultimately, samples were analyzed by LC-MS/MS, and data acquisition was conducted in data-dependent acquisition mode, as previously described^38^. Downstream protein profile analysis was conducted with the DEP package (version 1.18.0), with DEPs selected if absolute fold change (FC) was >1.2 and a *P* < 0.05^39^-^41^. Analyses of the functional enrichment of genes were done using the Gene Ontology (GO) database (https://geneontology.org/) and visualized using the “clusterprofiler” package (version 3.18.1) in R (R Institute for Statistical Computing, Vienna, Austria)^26^.

### 2.11 Synthesis and purification of recombinant human protein

*VDAC1* was cloned into a pCold I vector with a 6 His-tag fusion (Sangon, Shanghai, China). Reporter plasmids were transformed into the *Escherichia coli* BL21 strain and cultured under particular conditions^26^. Isopropyl β-D-1-thiogalactopyranoside was used to induce VDAC1 expression. Subsequently, bacteria were collected and fragmented, followed by centrifugation to isolate inclusion bodies. After undergoing denaturation, renaturation, and elution, the VDAC1 protein was obtained^26^. VDAC1 concentration was determined and recorded by Nanodrop 2000 (Thermo Fisher Scientific, USA). The integrity and purity of the purified protein were examined using SDS-PAGE stained with coomassie brilliant blue (CBB).

### 2.12 LC-MS/MS metabolomics analysis

Kidney tissues (100 mg) of male mice were grounded and resuspended with prechilled 80% methanol. Samples were incubated with a gradient of methanol (4 °C) and centrifuged prior to injection into the LC-MS/MS system for analysis^42^, ^43^. Ultra-high performance (UHP) LC-MS/MS analysis was conducted with a Vanquish UHPLC system (Thermo Fisher Scientific, Germany), which was coupled with an Orbitrap Q Exactive^TM^ ultra-high-field orbitrap mass spectrometer (Thermo Fisher Scientific, Germany). Samples were injected onto a Hypersil Goldcolumn using a certain linear gradient and flow rate. Q Exactive^TM^ HF mass spectrometer was run in positive/negative polarity mode^44^.

Raw data recorded by UHPLC-MS/MS was calculated with the Compound Discoverer 3.1 (Thermo Fisher Scientific) to capture peak alignment, peak selection, and quantitation for each metabolite, and to predict the molecular formula. Metabolite identification was then performed by comparing the high-resolution secondary spectral databases mzCloud (https://www.mzcloud.org/), mzVault, and MassList primary database search (library search). Statistical analyses were performed using R (version 3.4.3), Python (version 2.7.6) and CentOS (release 6.6). When data were non-normally distributed, they were standardized to obtain relative peak areas. Compounds with CVs of relative peak areas in quality control (QC) samples >30% were removed, and final metabolite identifications and relative quantification results were obtained^42^.

Principal components analysis (PCA) was used to distinguish the distribution trends of samples in the groups receiving Cel or DMSO, using metaX software, QC PCA, PCA, and partial least squares discriminant analysis (PLS-DA)^45^. Selection of differentially expressed metabolites (DEMs) mainly relied on the parameters of variable importance in the projection (VIP), FC, and *t*-test* P*-values^46^, with thresholds set at VIP >1.0, FC >1.5 or FC <0.667 and *P* <0.05^47^-^49^. Definition and analysis of DEMs and classification of annotation was based on the Human Metabolome Database (HMDB; https://hmdb.ca/)^15^.

### 2.13 Molecular docking

Three-dimensional (3D) Cel structure information was downloaded from PubChem (https://pubchem.ncbi.nlm.nih.gov/). Detail information of targeted proteins VDAC1 (6TIR), PC (7WTA), PKM2 (8G2E), and FASN (3TJM) were obtained from the Protein Data Bank (PDB). To describe the structures of ligand and protein, we used the AutoDock Tools (https://autodock.scripps.edu/). Patterns of interaction between Cel and targeted proteins were computed using AutoDock Vina and PyRx 0.8 (https://pyrx.sourceforge.io/). Ultimately, analysis and visualization of all docking data were carried out by PyMOL (https://pymol.org/2/)^26^.

### 2.14 Statistical analyses

Identified through a minimum of three separate investigations, data from all analyses were presented as the mean ± standard error of the mean (SEM), except where noted. One-way ANOVA was applied to analyze data between groups. Two-sample unpaired *t*-tests were also conducted. Prism 8.2 (GraphPad, La Jolla, CA, USA) was conducted to analyze statistical data.

## 3. Results

### 3.1 Cel induced acute kidney injury and apoptosis in mouse kidney cells

**Figure 1:**
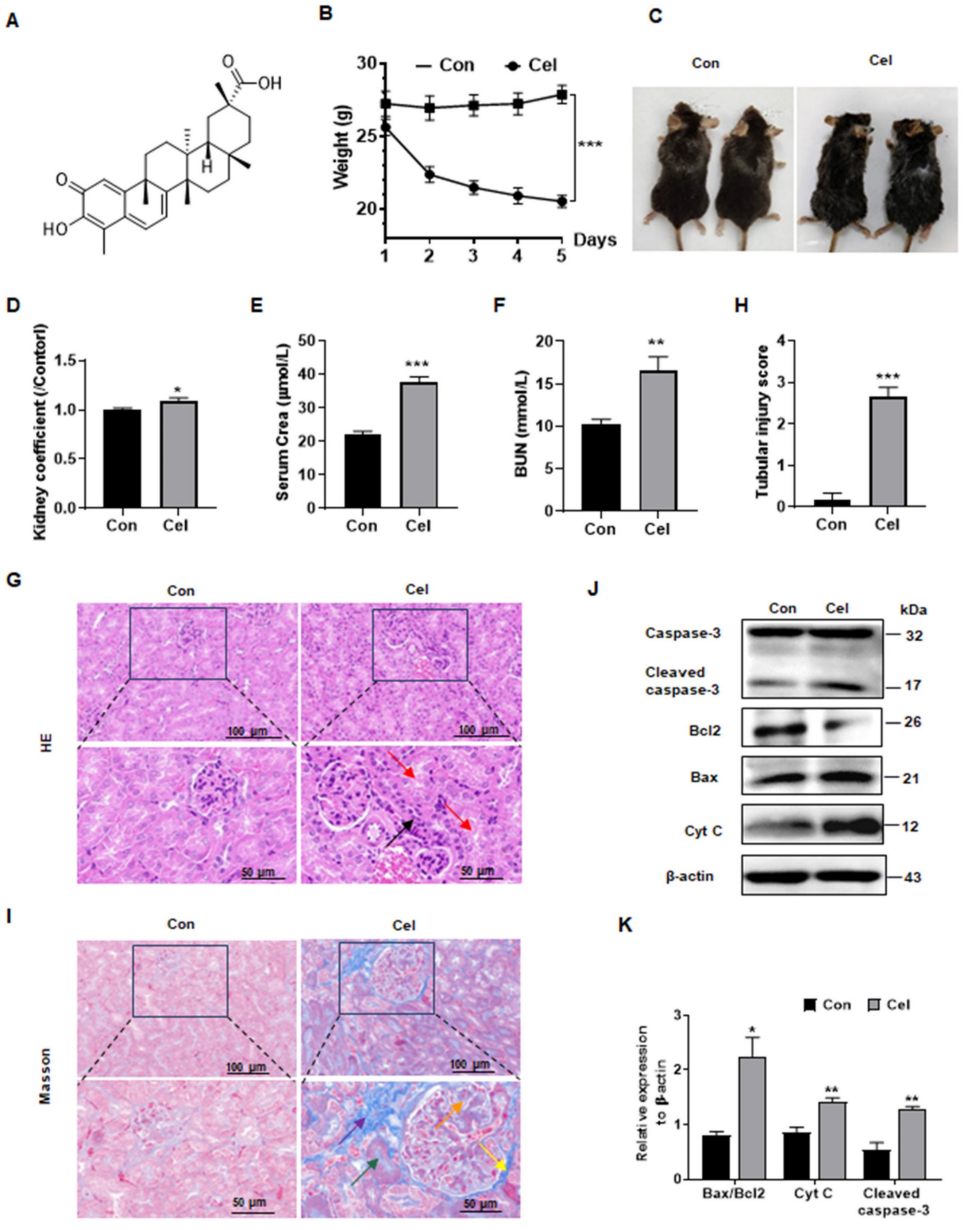
** Dysfunction and apoptosis of male mice kidney caused by Cel treatment.** (**A**) Cel chemical structure. (**B**) Bodyweight reduction was caused by Cel (5 mg/kg/d, i.p.) in male C57 mice (n = 6, *p* < 0.001). (**C**) Mouse appearance. (**D**) Cel increased mouse kidney coefficient (n = 6, *p* = 0.04). Cel treatment caused kidney dysfunction in mice, resulting in a significant increase in serum Crea (**E**, n = 4, *p* < 0.001), BUN (**F**, n = 6, *p* = 0.005). (**G**) H&E staining, (**H**) tubular injury score (n = 6, *p* < 0.001) and (**I**) Masson trichrome staining of male mouse kidney tissue. Scale bar = 50, 100 μm. (**J**) Expression and (**K**) corresponding statistical result of the proteins related to apoptosis in male mouse kidney tissue upon Cel treatment according to WB. Data were analyzed using unpaired two-tailed *t*-test with Prism 8.2 (mean ± SEM). ns, no significance, *p < 0.05, **p < 0.01, ***p < 0.001, *vs*. Control. Con, control, Cel celastrol, Crea creatinine, BUN blood urea nitrogen, H&E hematoxylin and eosin, WB western blotting, Cyt C cytochrome C, SEM standard error of the mean.

To investigate Cel nephrotoxicity (structural information as shown in Figure 1A)^27^, we conducted a series of* in vivo* experiments (Figure 1B-K). Cel administration significantly reduced C57 male mice bodyweight (Figure 1B) and caused cosmetic changes, including dehydration and loss of fur luster (Figure 1C). The kidney coefficient (kidneys weight/bodyweight) of male mice was increased upon Cel treatment (Figure 1D), suggesting an obvious toxic effect of Cel. Male mice in the Cel group exhibited severe kidney dysfunction with a significant increase in serum levels of Crea (Figure 1E) and BUN (Figure 1F). H&E staining (Figure 1G) demonstrated inflammatory cell infiltration in the renal interstitium (black arrow) and epithelial cell shedding in the tubular lumen, resulting in the formation of cellular debris (red arrow). The tubular injury score was significantly higher in the Cel group, suggesting kidney damage (Figure 1H). Fibrosis necrosis was observed by Masson's trichrome-staining (Figure 1I), including interstitial fibrosis (purple arrow), tubular fibrosis necrosis (green arrow), proliferation of glomerular thylakoid membrane (orange arrow) and basement membrane (yellow arrow).

Likewise, Cel caused significant bodyweight loss in female mice (Supplementary Figure 1A), and increased kidney coefficient (Supplementary Figure 1B). Cel treatment induced significant renal impairment, including elevated serum Crea (Supplementary Figure 1C) and BUN (Supplementary Figure 1D). Histological data from H&E staining (Supplementary Figure 1E) showed detachment of tubular endothelial cells (blue arrows), renal tubular vacuolization (black arrows), and a significant increase tubular injury score (Supplementary Figure 1F) in female mice with Cel treatment. Similar to the results of male mice, fibrosis of the tubular mesenchyme (red arrows), glomerular mesangium and glomerular basement membranes (yellow arrows) were found in female mouse in Masson staining (Supplementary Figure 1G).

It has been reported that some natural products cause cell apoptosis and induce toxic effects^14^, ^15^. Interestingly, as showed in Figure 1J and K: Cyt C expression and cleaved caspase-3 was significantly upregulated, and the Bax/Bcl2 ratio was increased, indicating kidney cell apoptosis. Cumulatively, these data showed that Cel induced obvious kidney damage and that kidney cell apoptosis may be related to mitochondria dysfunction.

### 3.2 ABPP strategy was used to identify Cel nephrotoxic targets

**Figure 2:**
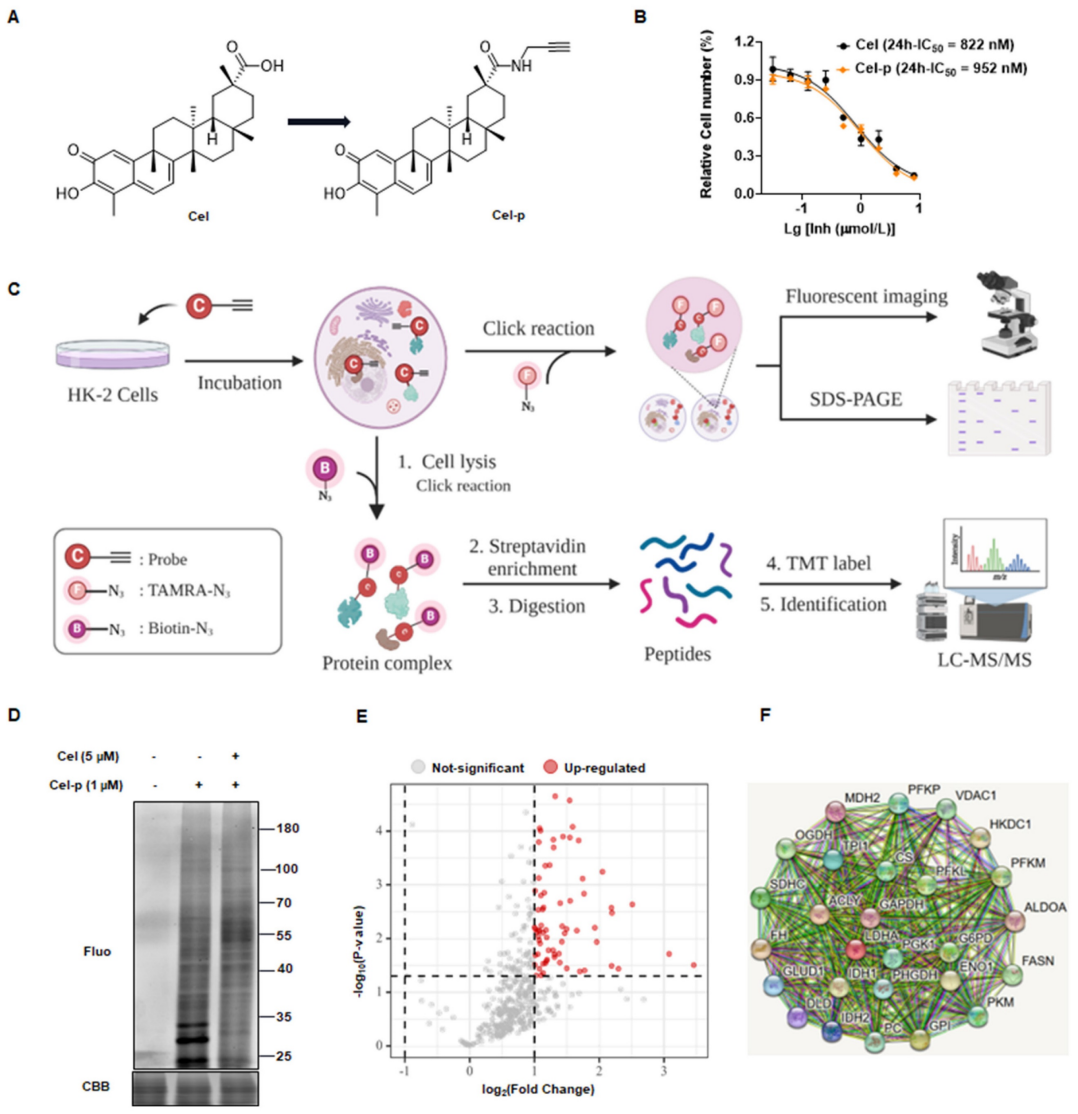
** Using ABPP method to identify Cel nephrotoxic targets.** (**A**) Cel-p chemical structure. (**B**) Toxicity of Cel and Cel-p on HK-2 cells. (**C**) Workflow of the strategy combining ABPP and click chemistry (schematic). This figure was created using www.biorender.com. (**D**) Excess Cel competed with labeled protein for binding to Cel-p *in situ*. (**E**) Volcano plot of the pulldown and identified proteins (red points) in Cel *vs.* DMSO groups by ABPP. (**F**) PPI network of target proteins (partial) of Cel bound to HK-2 cells. ABPP activity-based protein profiling, Cel celastrol, Cel-p celastrol probe, LC-MS/MS liquid chromatography-tandem mass spectrometry, TMT tandem mass tags, Fluo fluorescence, CBB coomassie brilliant blue, PPI protein-protein interaction.

To identify potential binding proteins of Cel in the kidney, we synthesized a Cel-p with the structural formula shown in Figure 2A. Cytotoxicities of Cel and Cel-p were highly similar (Figure 2B), suggesting the structural change of alkyne tag accession had little impact on Cel pharmacological activity. Therefore, Cel-p could be used to visualize and identify Cel targets using the ABPP method (Figure 2C). Results showed that plentiful proteins were labeled by probes, and fluorescence intensity increased in Cel-p concentration-dependent manner (Supplementary Figure 2A). Proteins labeled with Cel-p *in situ* were outcompeted by the co-incubation with excessive Cel (Figure 2D). As described above, the potent and selective binding targets (see red points) were pulled down by beads, enriched by biotin-azide and streptavidin, labeled with TMT reagent, and finally identified *via* LC-MS/MS analysis (Figure 2E). Moreover, analyses of signaling-pathway enrichment disclosed the biological processes (BPs) associated with these target proteins (Supplementary Figure 2B). Target proteins bound directly with Cel were involved in critical metabolic pathways, including the tricarboxylic acid (TCA) cycle (Supplementary Figure 2C). Notably, rate-limiting enzymes such as VDAC1, PC, PKM2, and FASN were highlighted in the protein-protein interaction (PPI) network according to the Search Tool for the Retrieval of Interacting Genes/Proteins (i.e., STRING) databases (https://cn.string-db.org/) (Figure 2F). Therefore, they were selected for Cel binding validation *in vivo* and *in vitro*.

### 3.3 Cel targets validated by combined PD-WB and CETSA-WB

**Figure 3:**
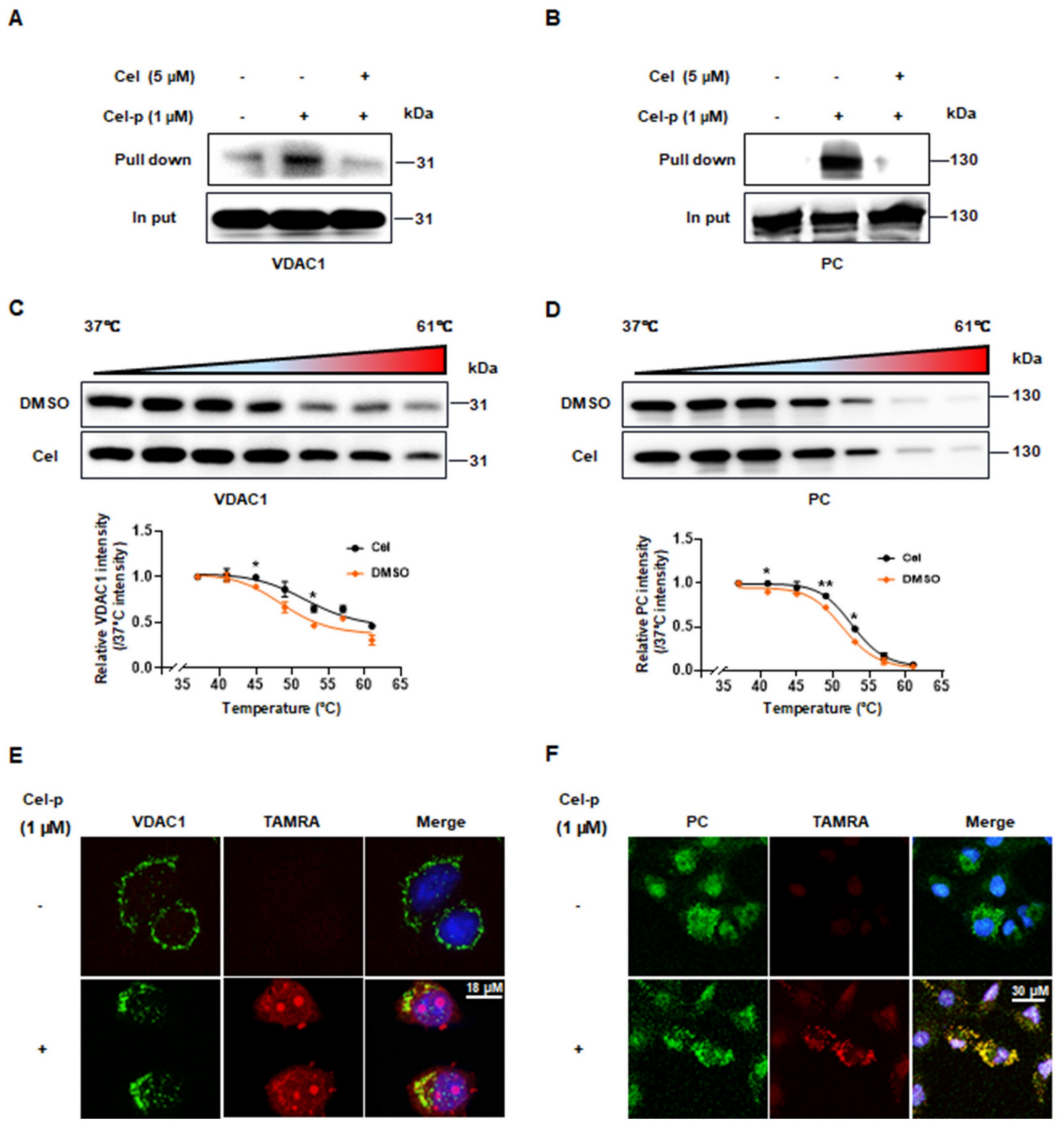
** Validation of Cel by combining PD-WB and CETSA-WB.** (**A** and** B**) PD-WB and input WB were carried out in kidney lysates to measure levels of VDAC1 and PC upon treatment with Cel-p and/or excess Cel. CETSA-WB from kidney lysate samples of VDAC1 (**C**, n =3, 45^o^C,* p* = 0.04; 53^o^C,* p* = 0.01) and PC (**D**, n =3, 41^o^C,* p* = 0.01; 49^o^C,* p* = 0.004; 53^o^C,* p* = 0.01) curves corresponding to the grayscale values of WB stripes were shown (repeated three times). Co-localization of VDAC1 (**E**) and PC (**F**) and Cel-p in cells was determined by fluorescence staining. Data were analyzed using an unpaired two-tailed *t*-test with Prism 8.2 (mean ± SEM). *p < 0.05, **p < 0.01, *vs*. Control. Cel celastrol, PD-WB, pull down-western blotting, CETSA-WB, cellular thermal shift assay Western blotting, Cel-p celastrol probe, VDAC1 voltage-dependent anion-selective channel protein 1, PC pyruvate carboxylase, SEM standard error of the mean.

PD-WB and CETSA-WB were conducted to further validate the target and mechanism of Cel nephrotoxicity. PD-WB results from HK-2 cell lysates showed that VDAC1 (Supplementary Figure 3A) and PKM2 (Supplementary Figure 3B) could be effectively pulled down, and that a high Cel dose could compete effectively with Cel-p. It also showed significant downward pulldown effects of PC (Supplementary Figure 3C) and FASN (Supplementary Figure 3D) with Cel-p, though the competition effect was slightly inferior. CETSA-WB of cells lysates showed that VDAC1 (Supplementary Figure 3E) and PC (Supplementary Figure 3F) exhibited similar thermal stability between groups, while it was higher in the Cel-treated group in PKM2 (Supplementary Figure 3G) and FASN (Supplementary Figure 3H). Their interaction was further indicated by results obtained from kidney tissue lysates. As expected, VDAC1 (Figure 3A) and PC (Figure 3B) were pulled down by Cel-p, and competed away by overdose of Cel. Moreover, VDAC1 (Figure 3C), PC (Figure 3D), PKM2 (Supplementary Figure 4A), and FASN (Supplementary Figure 4B) exhibited greater thermal stability in the Cel-treated group than in those treated with DMSO. Taken together, these results demonstrated that these proteins directly and stably bind with Cel.

Furthermore, immunofluorescence staining revealed that Cel co-localized with VDAC1 (Figure 3E), PC (Figure 3F), PKM2 (Supplementary Figure 4C), and FASN (Supplementary Figure 4D) in HK-2 cells. However, the pharmacological effects on expression of these proteins remain unknown, and thus require further investigation. WB results (Supplementary Figure 4E and F) showed a significant reduction of FASN but obvious increase of PKM2.

**Figure 4:**
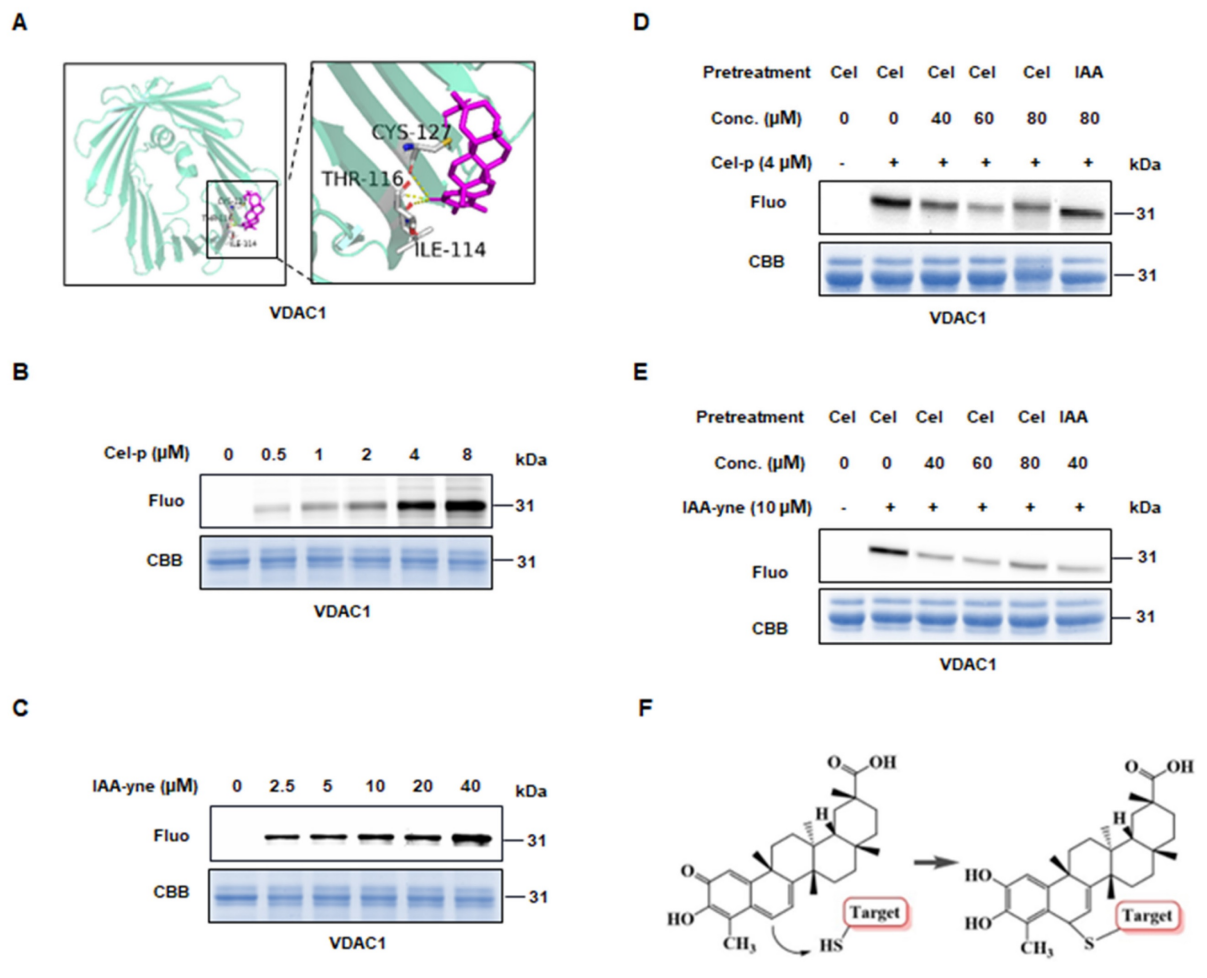
** Directly binding between Cel and VDAC1.** (**A**) Predicted binding sites of VDAC1 with Cel by Autodock. Yellow dotted lines represent hydrogen bonds, and purple sticks represent Cel compounds. (**B**) Cel-p and (**C**) IAA-yne labeling of recombined human VDAC1 protein (1-2 µg) in a dose-dependent manner. (**D**) Cel-p and (**E**) IAA-yne labeling of recombined human VDAC1 protein was done in the presence or absence of corresponding competitors. (**F**) Chemical changes of Cel upon binding with targets using a model presentation. Cel celastrol, IAA iodoacetamide, VDAC1 voltage-dependent anion-selective channel protein 1, Fluo fluorescence, CBB coomassie brilliant blue, Conc. concentration, IAA iodoacetamide, CYS cystine, THR threonine, ILE isoleucine.

To predict the potential binding sites of Cel targets, we conducted molecular docking using the resolved structure of proteins. Probably interrelations occurred between: ILE114, THR116, and CYS127 of VDAC1 with Cel (Figure 4A); CYS663, GLU664, LYS667, and GLU668 of PC (Supplementary Figure 5A); LYS162, ASN163, CYS165, LYS166, and LYS188 of PKM2 (Supplementary Figure 5B); and ASP2291, CYS2292, and LEU 2231 of FASN (Supplementary Figure 5C) as possibly bound to Cel. These results suggested that Cel targets specific cysteines in VDAC1, PC, PKM2, and FASN. Next, IAA (Cys-alkylator) was used to determine whether Cel binds targets through cysteines sites. Recombined VDAC1, purified by the methods described above, was co-incubated with probes, after which a click reaction using TAMRA-azide was conducted. The fluorescence intensity of the protein-probe binding was shown to be dose-dependent (Figure 4B and C), indicating that Cel combined VDAC1 by covalent means. Then, VDAC1 protein was pretreated with an overdose of Cel or IAA, incubated with probes, and subjected to a click reaction. Cel-p was competed away by Cel and IAA, despite its slightly weaker effectiveness (Figure 4D and E). Change of Cel chemical structure after binding targets was demonstrated (Figure 4F). These data implied that cysteine residue may be the Cel-VDAC1 binding group. These cumulative findings reveal that Cel induced kidney dysfunction by targeting multiple crucial metabolic enzymes, and that cysteine residue is the key Cel target-induced nephrotoxicity binding group.

### 3.4 Proteomics analysis revealed *in vitro* Cel impaired mitochondrial function

**Figure 5:**
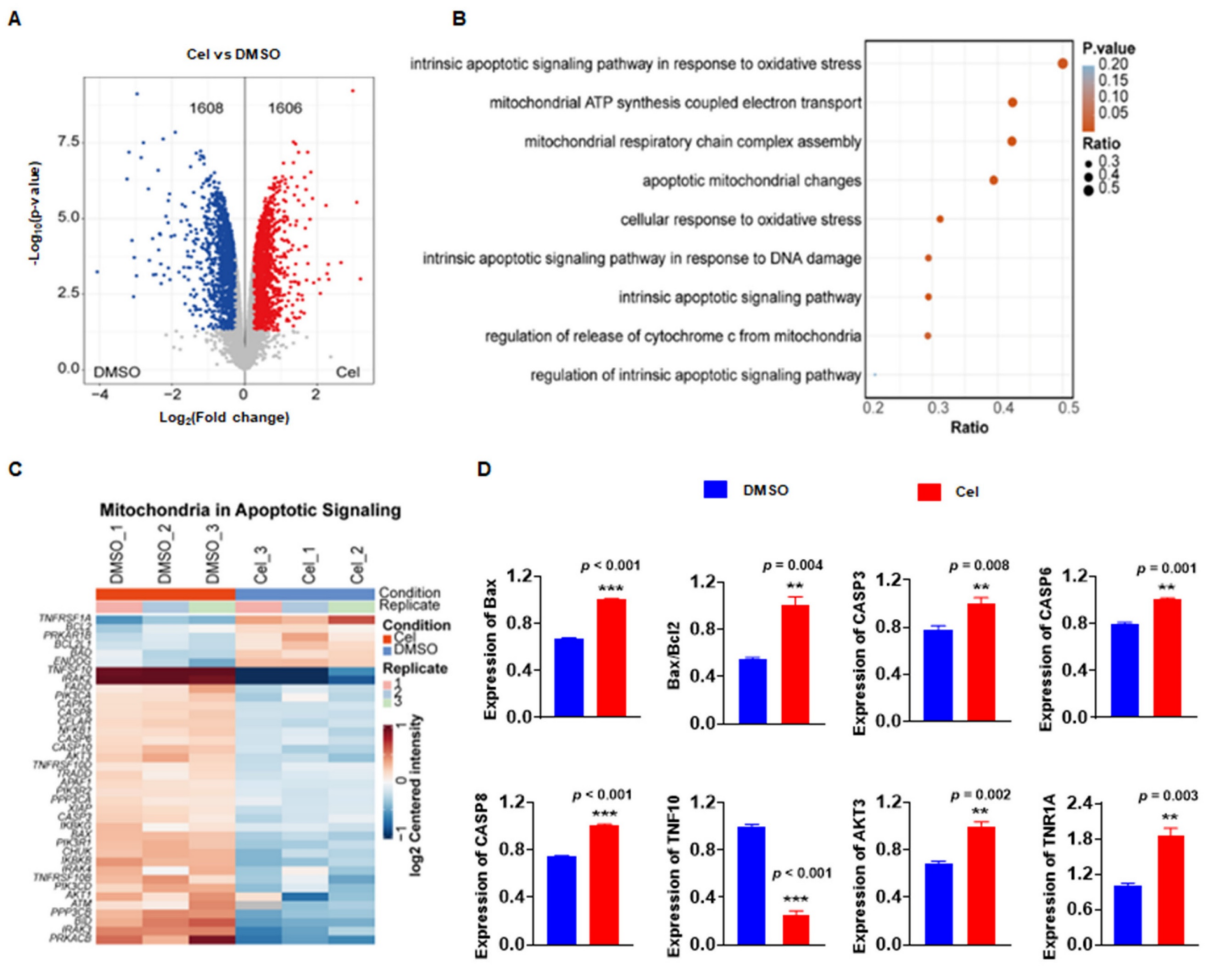
** Proteomics analysis demonstrated that Cel caused mitochondrial dysfunction and apoptosis *in vitro.*** (**A**) Volcano plot depicting DEPs in Cel *vs*. Control (DMSO) groups. (**B**) Bubble diagram illustrating the analysis of signaling-pathway enrichment for DEPs using the KEGG database. (**C**) Cluster analysis of DEPs. (**D**) Statistical analyses of apoptosis related proteins among DEPs. Data were calculated by unpaired two-tailed *t*-test (mean ± SEM, n = 3). **p < 0.01, ***p < 0.001, *vs*. Control. Cel celastrol, DEPs differentially expressed proteins, SEM standard error of the mean.

Cel-induced nephrotoxicity targets have been identified and confirmed by ABPP, CETSA-WB, PD-WB, and other methods. However, the potential molecular signaling pathways and BPs were unknown. Thus, a proteomics analysis of cell samples was undertaken. Compared with the DMSO group, the Cel-treated group exhibited 1608 proteins with downregulated expression and 1606 proteins with upregulated expression (Figure 5A). Analyses of signaling pathways showed that the most pathway-enriched DEPs were associated with mitochondrial metabolism, oxidative stress, and apoptosis (Figure 5B). Cluster analysis was used to assess mitochondrial apoptotic signaling of the specific DEPs (Figure 5C). These proteins were also quantified, and the expression of most was significantly upregulated with Cel treatment, including caspase-3, Bax/Bcl2, caspase-6, and AKT3 (Figure 5D). Indeed, our results from WB of tissue lysates (Figure 1J and K) confirmed that Cel induced mitochondria-related apoptosis *in vivo*.

Taken together, proteomics analysis showed that Cel can damage kidney cells through BPs related to mitochondrial metabolism, oxidative stress, and apoptosis.

### 3.5 Cel reduced MMP and aggravated apoptosis *in vitro*

**Figure 6:**
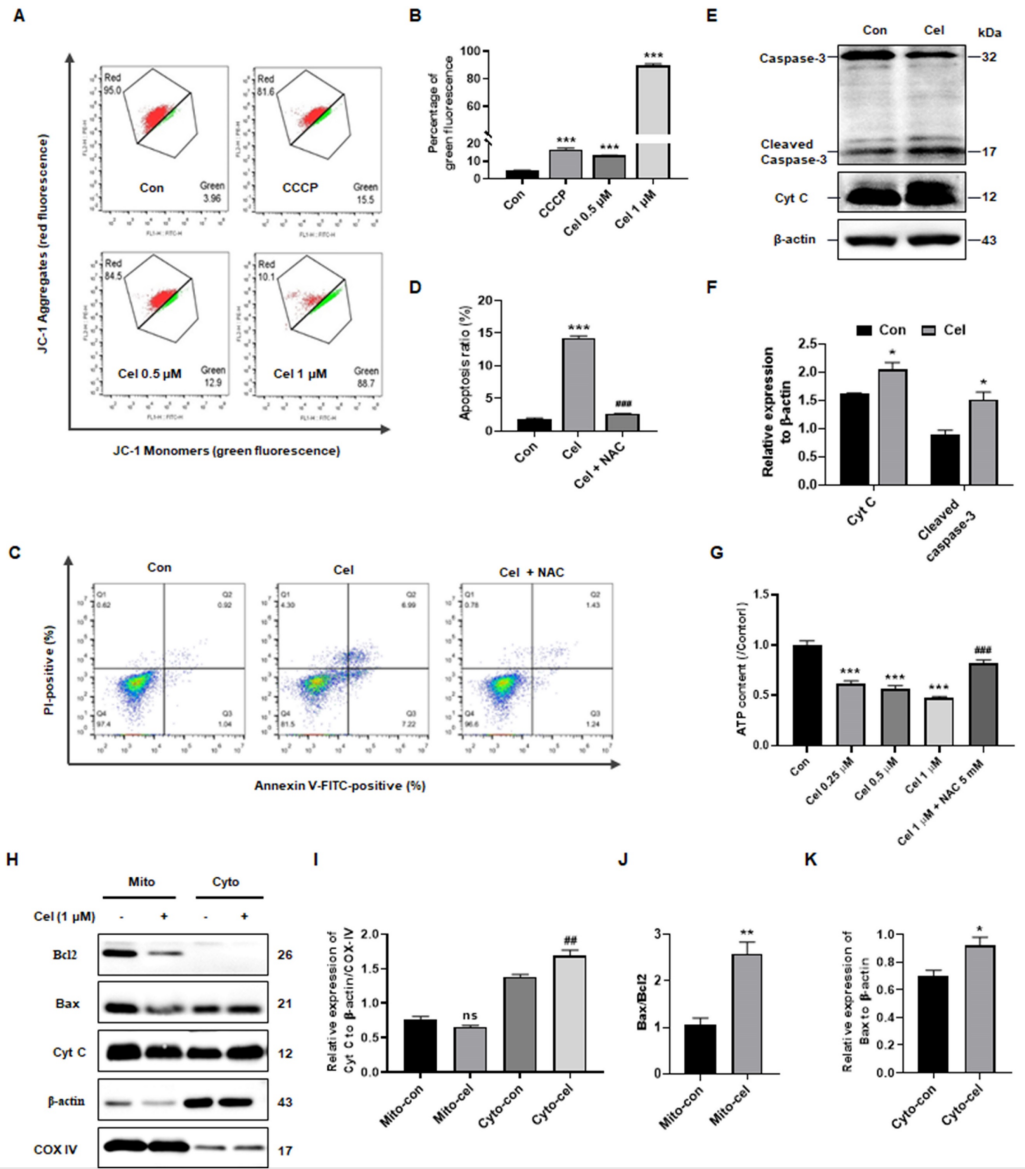
** Cel reduced MMP and induced apoptosis in HK-2 cells.** (**A**) JC-1 monomers of HK-2 cells were measured and (**B**) statistical analyses for different concentrations of Cel (n = 3, *p* < 0.001). (**C** and** D**) The apoptosis of cells was detected with Cel treatment or NAC pretreatment (5 mM) (n = 3, *p* < 0.001). (**E** and **F**) Expression of apoptosis-related proteins was measured by WB (n = 3, Cyt C, *p* = 0.02; cleaved caspase-3, *p* = 0.02). (**G**) ATP content of HK-2 cells with Cel treatment (n = 3, *p* < 0.001, ###*p* < 0.001 for Cel + NAC *vs*. Cel). (**H**) The WB assay was detected in mitochondria and cytosol isolated from male mice kidneys. (**I**) The expression of Cyt C in mitochondria and in cytosol (n = 3, ##*p* = 0.007, cyto-cel *vs*. cyto-con). (**J**) The ratio of Bax/Bcl2 in mitochondria (n = 3, **p = 0.006, mito-cel *vs*. mito-con). (**K**) The expression of Bax in cytosol (n = 3, *p = 0.04, cyto-cel *vs*. cyto-con). Data in B, D, and G were analyzed using one-way ANOVA, and data in F, I, J, and K were used unpaired two-tailed *t*-test with Prism 8.2 (mean ± SEM). ns, no significance, *p < 0.05, ***p < 0.001, *vs*. Control. Cel celastrol, MMP mitochondrial membrane potential, NAC N-Acetyl-L-cysteine, ATP, adenosine triphosphate, WB western blotting, SEM standard error of the mean.

Based on data from kidney tissues (Figure 1J and K) and proteomics analysis of cell samples (Figure 5A-D), Cel treatment was found to impact the expression of genes related to apoptosis, particularly those associated with mitochondria. Flow cytometry was then conducted to further investigate alterations in mitochondria. JC-1 detection revealed that Cel reduced MMP of cells in a concentration-dependent manner, indicating an early stage of apoptosis (Figure 6A and B). The pro-apoptotic effects of Cel were largely blocked by NAC (Figure 6C and D). WB results confirmed a significant release of Cyt C and cleaved caspase-3 induced by Cel treatment in HK-2 cells (Figure 6E and F). Furthermore, ATP levels reduced in a concentration-dependent manner after 24 h with Cel administration, but pretreatment with NAC (2 h) rescued this decrease (Figure 6G). WB analysis of mitochondria isolated from mouse tissues (Figure 6H) showed a slight decrease in mitochondrial Cyt C expression in the Cel group, but an obvious up-regulation in the cytosol group (Figure 6I). Additionally, the Bax/Bcl2 ratio in mitochondria was significantly higher in the Cel group compared with the control group (Figure 6J), while Bax expression in the cytosol group was also significantly increased following Cel treatment (Figure 6K).

In summary, Cel treatment induced nephrotoxicity through dysfunction in ATP metabolism and mitochondria-dominated apoptosis.

### 3.6 Nontargeted metabolomics analysis identified the primary metabolic pathways involved in Cel nephrotoxicity

**Figure 7:**
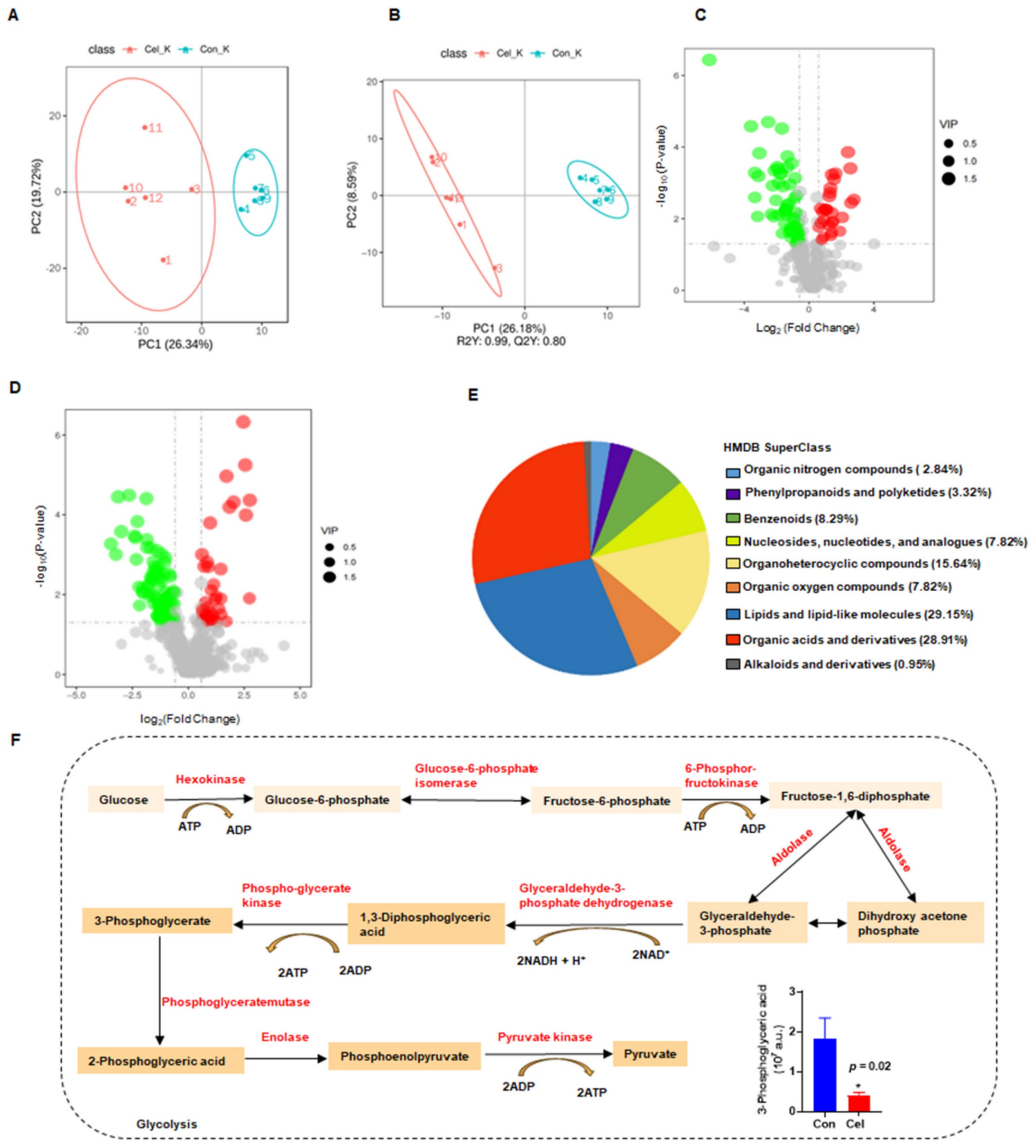
** Non-targeted metabolomics analysis revealed the main processes involved in Cel induced-nephrotoxicity.** (**A**) PCA in NIM. (**B**) Neg PLS-DA score plots of DMSO and Cel groups in kidney tissue. (**C** and **D**) Volcano plots showing DEMs in NIM and PIM. (**E**) Classification annotations (based on HIMDB database) in the kidney following Cel-treatment. (**F**) Cel regulated the expression of rate-limiting enzymes (highlighted in red) and their corresponding products or substrates (n = 6). Data were analyzed using an unpaired two-tailed *t*-test with Prism 8.2 (mean ± SEM). *p < 0.05 *vs*. Control. Cel celastrol, Neg PLS-DA negative partial least squares-discriminant analysis, DEMs differentially expressed metabolites, PIM positive ion mode, NIM negative ion mode, HMDB human metabolome database, SEM standard error of the mean.

UHPLC-MS/MS was used to analyze kidney tissue and characterize Cel nephrotoxicity. First, the QC of kidney samples was high in terms of their intercorrelations, both of negative ion mode (NIM, Supplementary Figure 6A) and positive ion mode (PIM, Supplementary Figure 6B). Similar results were observed from QC PCA analysis of NIM (Supplementary Figure 6C) and PIM (Supplementary Figure 6D), evidenced by the distribution of QC samples (blue points) clustering together. These results indicated that the quality and stability of samples during analyses were reliable in both ion modes [50]. Additionally, PCA of NIM (Figure 7A) indicated a significant distribution of metabolites with- or without-Cel treatment groups.

Finally, a total of 213 DEMs with obvious differences were detected, including 87 DEMs in NIM and 126 DEMs in PIM. The negative PLS-DA (neg PLS-DA) score was notably different between the DMSO and Cel groups (Figure 7B), with similar results observed in the positive PLS-DA (Supplementary Figure 6E). The metabolites in NIM were classified and determined to be primarily “lipids and lipidlike molecules” (Supplementary Figure 6F). Additionally, as shown in Figure 7C, 28 DEMs had up-regulated expression (red points) and 59 DEMs had downregulated expression (green points) in NIM. While in PIM (Figure 7D), 40 DEMs with upregulated expression (red points) and 86 DEMs with downregulated expression (green points,) were found. Next, we analyzed metabolites from the two ion modes based on the HMDB. The metabolites that changed under Cel treatment were primarily “lipids and lipid-like molecules” (Figure 7E). Cel affected almost all the rate-limiting enzymes associated with the glycolysis pathway (highlighted in red in Figure 7F), resulting in a decrease in the production of 3-phosphoglyceric acid.

In summary, metabolomics analysis suggested that Cel damages critical metabolic processes, including glycolysis, by affecting multiple key enzymes involved in complex molecular patterns.

## 4. Discussion

**Figure 8:**
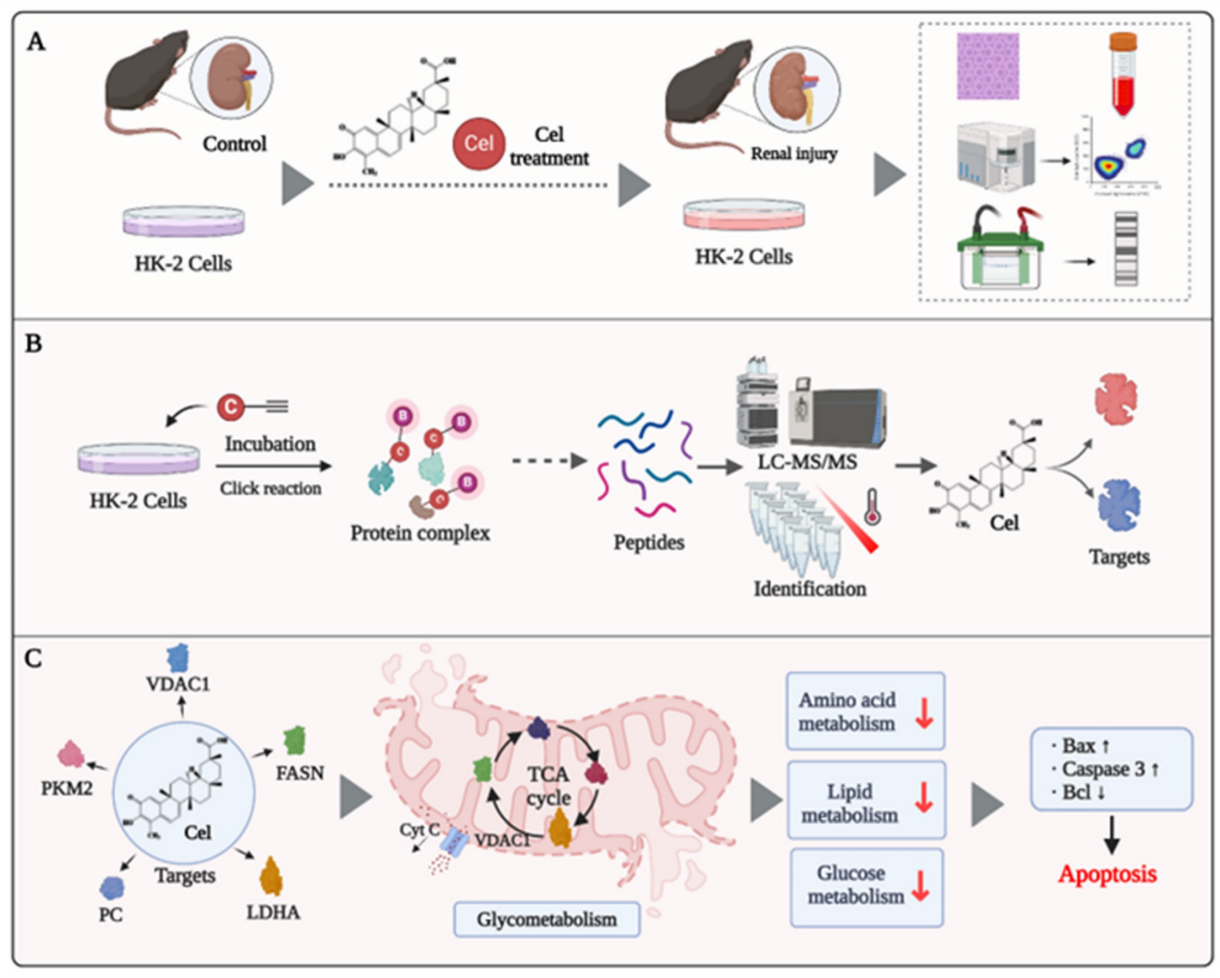
** Potential mechanism of Cel-induced nephrotoxicity (schematic).** (**A**) Cel sources and its nephrotoxicity *in vivo* and *in vitro*. (**B**) Strategy combining ABPP and CETSA to identify and validate the binding targets of Cel on HK-2 cells and kidney tissues. (**C**) Cel targeted the proteins (enzymes) associated specifically with metabolism, ultimately resulting in mitochondrial dysfunction and apoptosis. This figure was drawn using www.biorender.com. Cel celastrol, ABPP activity-based protein profiling, CETSA cellular thermal shift assay.

In recent decades, Cel has been shown to have pharmacological activities, including sensitizing leptin, anti-inflammatory effects, and anti-tumor properties. These features make it a promising drug for the treatment of diabetes mellitus, lupus nephritis, and cancer^51^, ^52^. However, limited understanding of its toxicity (particularly nephrotoxicity) hinders Cel development and application. Herein, we achieved a deeper understanding of the mechanisms underlying Cel nephrotoxicity by integrating ABPP, CETSA, proteomics, and metabolomics analysis. Multiple and complex mechanisms (e.g., regulation of enzymes participating in the TCA cycle and glycolysis) were involved. Cel treatment caused mitochondrial dysfunction and apoptosis in kidney cells (Figure 8). Our study thus provides invaluable evidence for preventing the adverse effects of Cel and developing drugs to counteract its nephrotoxicity, thereby facilitating expansion of its clinical applications.

*In vivo*, Cel generated acute damage to kidney function in mice, resulting in a sharp rise in levels of Crea, BUN, and other renal function indices. Histology revealed clear vacuolization, fibrosis, and necrosis of renal tubular cells, along with glomerular hyperplasia and fibrotic degeneration. Furthermore, in kidney tissue and cell lysates, expression of Bax/Bcl2, CytC, and caspase-3 was upregulated significantly, indicating exacerbated apoptosis and kidney tissue damage following Cel treatment (Figure 8A). These effects were alike nephropathy influenced by aristolochic acid^15^. Previous studies have indicated that drugs with an IC_50_ <1 µM can exhibit potent inhibition on multiple ion channels^11^. Accordingly, our data suggests that Cel impaired VDAC1 expression, thereby disrupting the relevant functions of mitochondria and inducing kidney cell apoptosis or necrosis. The JC-1 assay also showed that Cel diminished the MMP and caused apoptosis. Combining TMT and metabolomics analysis revealed that Cel regulated various key enzymes related to energy metabolism. CETSA-WB and PD-WB further confirmed the specific damaging effect of Cel on VDAC1, PC, PKM2, and FASN (Figure 8B). Cel overdose downregulated several crucial components of the TCA cycle and glycolysis resulting in mitochondrial metabolic disorders and apoptosis (Figure 8C).

It was confirmed that PKM2 expression is up-regulated under stimuli such as toxicity studies, or oxidative stress^53^. Similarly, WB of kidney lysate showed that PKM2 expression increased with treatment of Cel. In line with Cao et al., it was observed that FASN expression was significantly downregulated in the Cel group, suggesting that Cel treatment inhibited lipid metabolism^54^. Clarification of the specific mechanism of these results will need further investigation.

Mitochondrial structural integrity is essential for their function and maintenance of intracellular homeostasis. Indeed, drug toxicity can promote apoptosis by disrupting the integrity of the mitochondrial membrane and the internal structure of cells^15^, ^55^. Impairment of mitochondrial function is closely associated with the development of acute kidney injury^56^, diabetic kidney disease^57^, and chronic kidney disease^58^. Our study confirmed that Cel causes dysfunction and apoptosis of mitochondria. This was verified including by WB experiment of isolation from mitochondria and cytosol, JC-1 test, and ATP content detected. Furthermore, as the 'gatekeeper' of mitochondria, VDAC1 is an essential component of the outer mitochondrial membrane (OMM). It plays a crucial part in regulating vital physiological processes, such as metabolism and apoptosis^59^. We discovered that Cel decreased the MMP, implying damage to the OMM. Studies have shown that if VDAC1 is overexpressed, it undergoes a transition to an oligomeric state, leading to macropore formation^60^. These macropores increase the OMM permeability, facilitating release of apoptosis-related proteins (e.g., Cyt C) into the cytoplasm^60^, ^61^. VDAC1 oligomerization is a feature of the early stage of apoptosis^61^-^63^. The corresponding cascade reaction is subsequently activated, including caspase family proteins that induce apoptosis. Moreover, VDAC1 interacts with BAX family proteins, such as Bax and Bcl2, to promote the apoptotic program^60^, ^61^, which was validated by the results of WB and proteomics analyses herein. Moreover, VDAC1 participates in substrate metabolism, including the TCA cycle, glycolysis, and lipid metabolism^63^, ^64^. These functions may be undertaken by regulating calcium ions in mitochondria, as well as substances such as pyruvate in the cytoplasm^65^, ^66^. Therefore, Cel caused nephrotoxicity *via* complex pathways involved in important mitochondrial BPs.

Though our findings further identify the covalent targets of Cel, these experimental methods were insufficient to identify the non-covalent or reversible binding proteins of Cel. In addition, while we focused on the mechanisms of Cel nephrotoxicity in the hyperacute phase, we did not investigate subacute or prolonged Cel nephrotoxicity. Moreover, that a substantial portion of *in vivo* data was obtained from male mice, which may be a limitation as we were unable to determine sex differences in the mechanism of Cel-induced nephrotoxicity. These information gaps should be further explored.

In summary, we have reported herein, for the first time, that the nephrotoxicity elicited by Cel may primarily occur by targeting mitochondrial proteins and inducing mitochondrial dysfunction, which ultimately leads to apoptosis.

## 5. Conclusion

By combining ABPP, CETSA, metabolomics, and proteomics analyses, we discovered that Cel directly targets critical enzymes involved in the TCA cycle, glycolysis, lipid metabolism, and amino acid metabolism. Cel treatment led to mitochondrial dysfunction, ultimately resulting in kidney cell apoptosis. Our findings provide new insights into the mechanism of Cel nephrotoxicity.

## Supplementary Material

Supplementary figures.

## Abbreviations

Cel: Celastrol
ABPP: activity-based protein profiling
PKM2: pyruvate kinase 2
Cel-p: Cel-probe
CETSA: cellular thermal shift assay
CETSA-WB: CETSA western blotting
PD-WB: pull down-western blotting
DMSO: deferoxamine mesylate
CCK8: Cell counting Kit-8
NAC: N-Acetyl-L-cysteine
THPTA: Tris (3-hydroxypropyltriazolylmethyl) amine
NaVc: vitamin C sodium
LC-MS/MS: liquid chromatography-tandem mass spectrometry
TMT: tandem mass tags
IAA: iodoacetamide
Cyt C: cytochrome C
PC: pyruvate carboxylase
FASN: fatty acid synthase
VDAC1: voltage-dependent anion-selective channel protein 1
HK-2: human renal tubular epithelial cell line
PFA: paraformaldehyde
i.p.: intraperitoneally
Crea: creatinine
BUN: blood urea nitrogen
H&E: hematoxylin and eosin
MMP: mitochondrial membrane potential
ATP: adenosine triphosphate
PBS: phosphate-buffered saline
SDS: sodium dodecyl sulfate
PAGE: polyacrylamide gel electrophoresis
RT: room temperature
DEPs: differentially expressed proteins
FC: fold change
GO: gene ontology
CBB: coomassie brilliant blue
UHP: ultra-high performance
QC: quality control
PCA: principal components analysis
PLS-DA: partial least squares discriminant analysis
DEMs: differentially expressed metabolites
VIP: variable importance in the projection
HMDB: human metabolome database
3D: there-dimensional
PDB: Protein Data Bank
SEM: standard error of the mean
BPs: biological processes
TCA: tricarboxylic acid
PPI: protein-protein interaction
STRING: Search Tool for the Retrieval of Interacting Genes/Proteins
Conc.: concentration
CYS: cystine
THR: threonine
ILE: isoleucine
CYS: cystine
GLU: glutamic acid
LYS: lysine
ASN: asparagine
LEU: leucine
NIM: negative ion mode
PIM: positive ion mode
OMM: outer mitochondrial membrane
Con: control
ns: no significance
