# Supplementary material for: Dissection of Targeting Molecular Mechanisms of Celastrol-induced Nephrotoxicity via A Combined Deconvolution Strategy of Chemoproteomics and Metabolomics
Source: Int J Biol Sci. 2024 Aug 26;20(12):4601–17. doi: 10.7150/ijbs.91751 (PMC11414378; doi:10.7150/ijbs.91751)
Supplement: Supplementary file 1 — Supplementary figures. [file ijbsv20p4601s1.pdf]

Dissection of Targeting Molecular Mechanisms of Celastrol-induced Nephrotoxicity *via* A Combined Deconvolution Strategy of Chemoproteomics and Metabolomics

Xueying Liu [a, c, #], Qian Zhang [a, b, #], Peili Wang [a, g, #], Xin Peng [a, f, #], Yehai An[a, b], Junhui Chen[a], Jingnan Huang[a], Shuanglin Qin [h], Hengkai He [a], Mingjing Hao [a], Jiahang Tian [b], Letai Yi [a, d], Ming Lei [a, i, *], Piao Luo [a, b, h, *], Jigang Wang [a, b, e, f, *], Xinzhou Zhang [a, c, *]

Supplementary materials

Dissection of targeting molecular mechanisms of celastrol-induced nephrotoxicity *via* a combined deconvolution strategy of chemoproteomics and metabolomics

The followings are the Supplementary materials to this article.

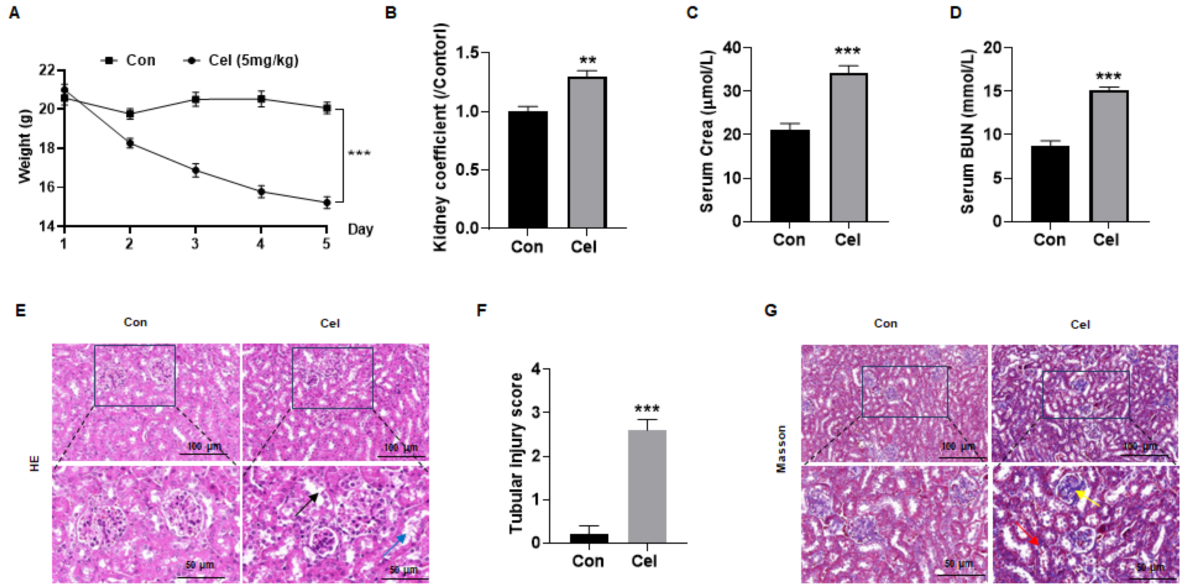

Supplementary Figure 1. Cel caused kidney injury in female mice and mitochondrial apoptosis.

(**A**) Cel treatment reduced the bodyweight in C57 female mice (n = 5, $p < 0.001$). (**B**) Kidney coefficient of mice was increased with Cel treatment (n = 5, $p = 0.003$). A significant increase in serum levels of Crea (**C**, n = 4 $p < 0.001$), BUN (**D**, n = 5, $p < 0.001$) was observed in Cel group. (**E**) H&E staining of kidney tissue. (**F**) The tubular injure score of female mice (n = 5, $p < 0.001$). (**G**) Masson staining of kidney. Scale bar = 50, 100 μm. Data were analyzed using an unpaired two-tailed *t*-test (mean ± SEM) *$p < 0.05$, ***$p < 0.001$, *vs*. Control. Cel celastrol, Crea creatinine, BUN blood urea nitrogen, H&E hematoxylin and eosin, SEM standard error of the mean.

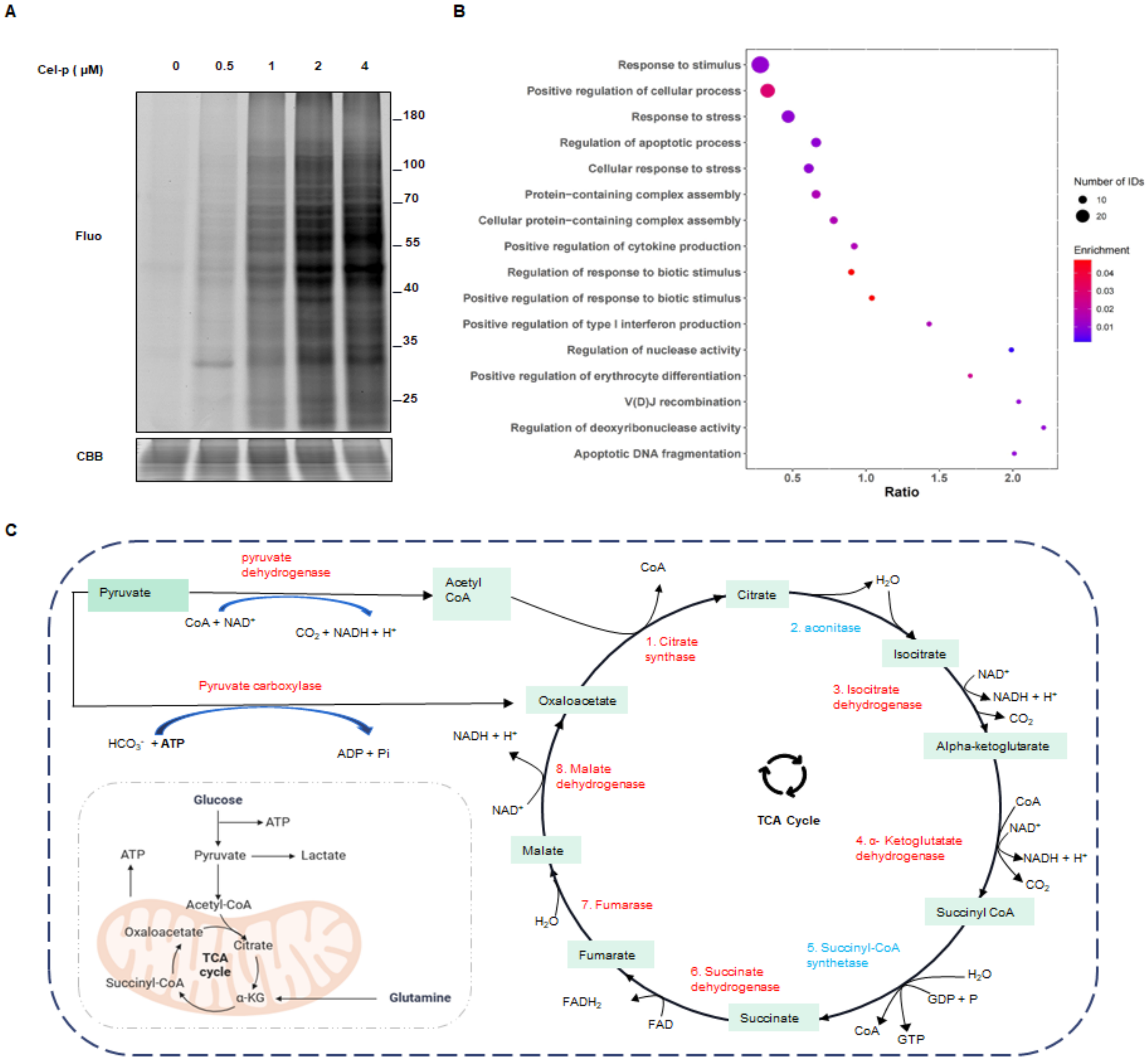

Supplementary Figure 2. Targeted specific proteins of Cel on HK-2 cells.

(**A**) Proteins were labeled in a dose-dependent manner on HK-2 cells *in situ* by Cel-p. (**B**) Bubble diagram illustrating the enrichment of BPs from proteins pulled down using the TMT assay. (**C**) Cel regulated the TCA cycle by influencing crucial enzymes (highlighted in red). Cel celastrol, Cel-p celastrol probe, BPs biological processes, TMT tandem mass tags, Fluo fluorescence, CBB coomassie brilliant blue.

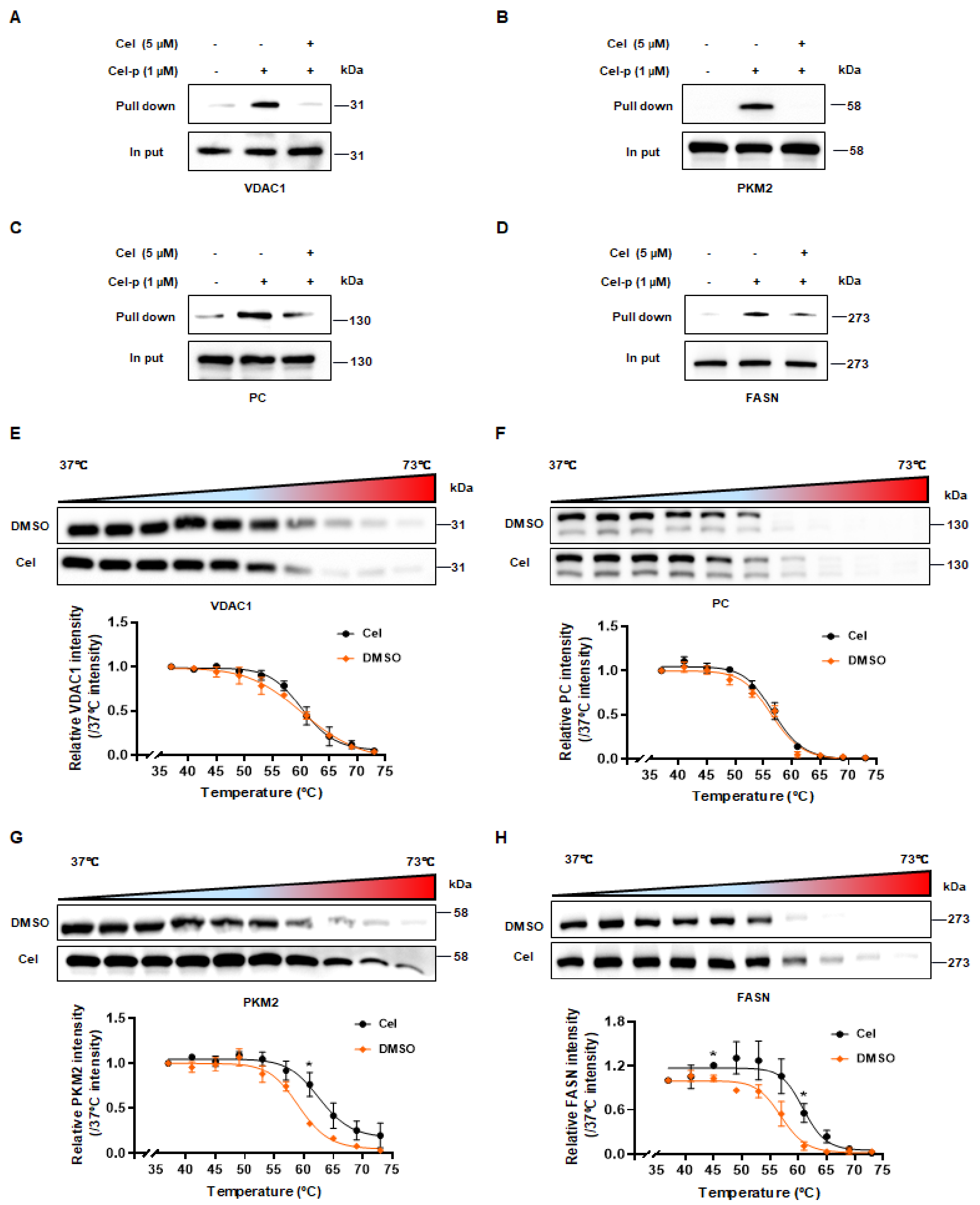

Supplementary Figure 3. Validation of Cel targeted-proteins by combining PD-WB and CETSA-WB of HK-2 cells.

PD-WB and input WB were conducted to detect the proteins of VDAC1 (**A**), PKM2 (**B**), PC (**C**), and FASN (**D**) with treatment of Cel-p and/or excess dose of Cel upon cell lysates. Stripes and curves corresponding to the grayscale values of CETSA-WB of VDAC1 (**E**), PC (**F**), PKM2 (**G**, 61°C, p = 0.03), and FASN (**H**, 45°C, p = 0.02, 61°C, p = 0.03) were displayed.

Data were analyzed using an unpaired two-tailed *t*-test (mean ± SEM, n = 3). *$p < 0.05$, *vs*. Control. PD-WB pull down western blotting, CETSA cellular thermal shift assay, PKM2 pyruvate kinase 2, FASN fatty acid synthase, PC pyruvate carboxylase, VDAC1 voltage-dependent anion-selective channel protein 1, Cel-p celastrol probe, SEM, standard error of the mean.

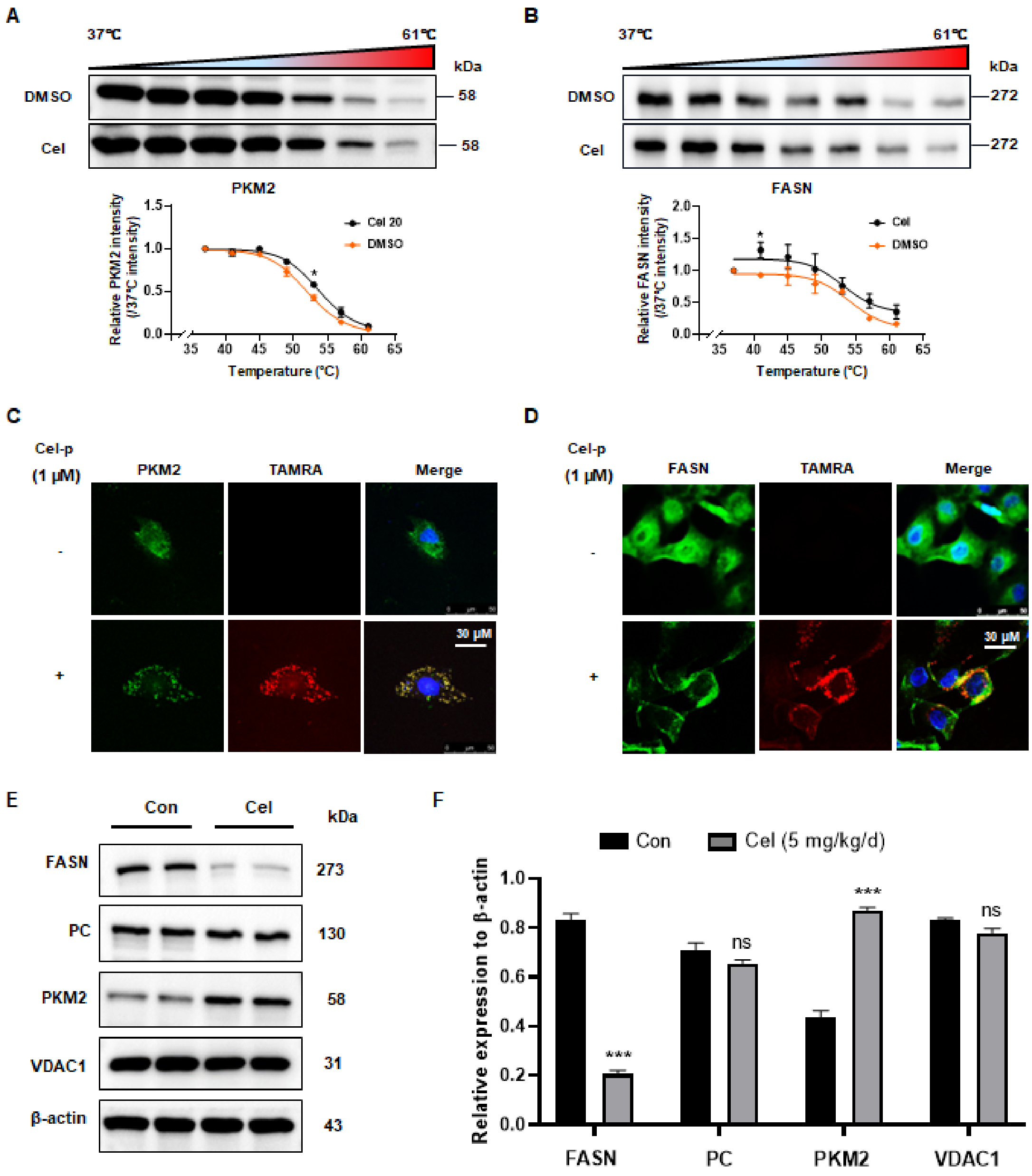

Supplementary Figure 4. Validation of targeted proteins with CETSA and their co-localization.

(**A** and **B**) Validation of targets PKM2 (n = 3, 53°C, p =0.04) and FASN (41°C, p =0.02) was performed using CETSA-WB in male mice kidney samples. Curves corresponded to the grayscale values of WB stripes, which were measured three times. (**C** and **D**) Co-localization of protein-targeted PKM2 and FASN proteins with Cel-p using fluorescence staining. Scale bar = 30 μm. (**E**) The expression and corresponding statistics (**F**) of targeted-proteins with treatment of Cel or corn oil in male mice kidney. Data were analyzed using an unpaired two-tailed *t*-test (mean ± SEM, n = 3). ns, no significance, *p < 0.05, ***p < 0.001 *vs*. Control. CETSA cellular thermal shift assay, PKM2 pyruvate kinase 2, FASN fatty acid synthase, PC pyruvate carboxylase, VDAC1 voltage-dependent anion-selective channel protein 1, WB western blotting, Cel-p celastrol probe, SEM, standard error of the mean.

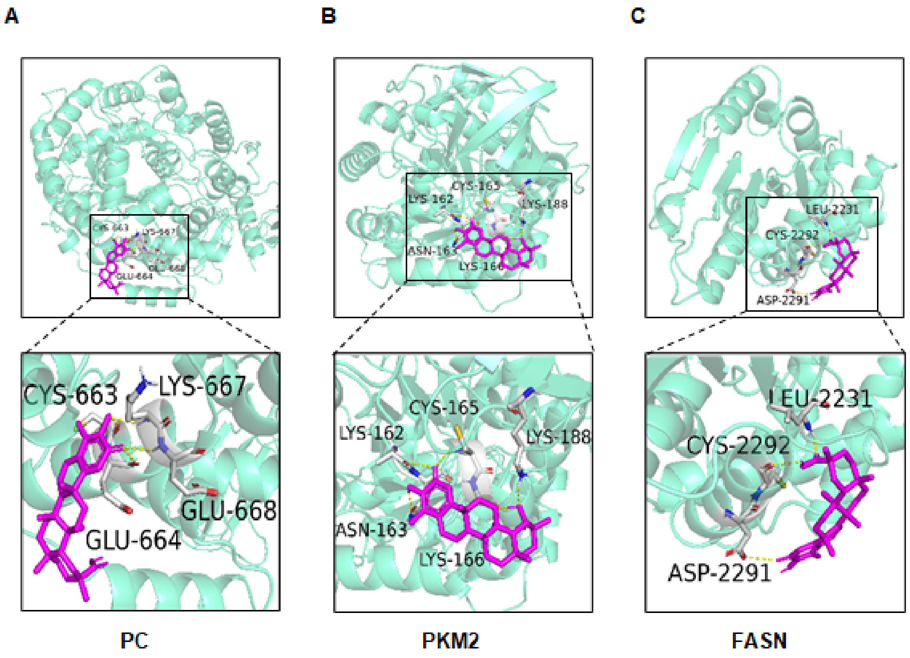

Supplementary Figure 5. Predicted binding sites of Cel with target proteins by Autodock.

(**A-C**) Binding model of Cel with PC, PKM2, and FASN through molecular docking. Yellow dotted lines represent hydrogen bonds, and purple sticks represent the compounds of Cel. Cel celastrol, PC pyruvate carboxylase, PKM2 pyruvate kinase 2, FASN fatty acid synthase, CYS cystine, GLU glutamic acid, LYS lysine, ASN asparagine, LEU leucine.

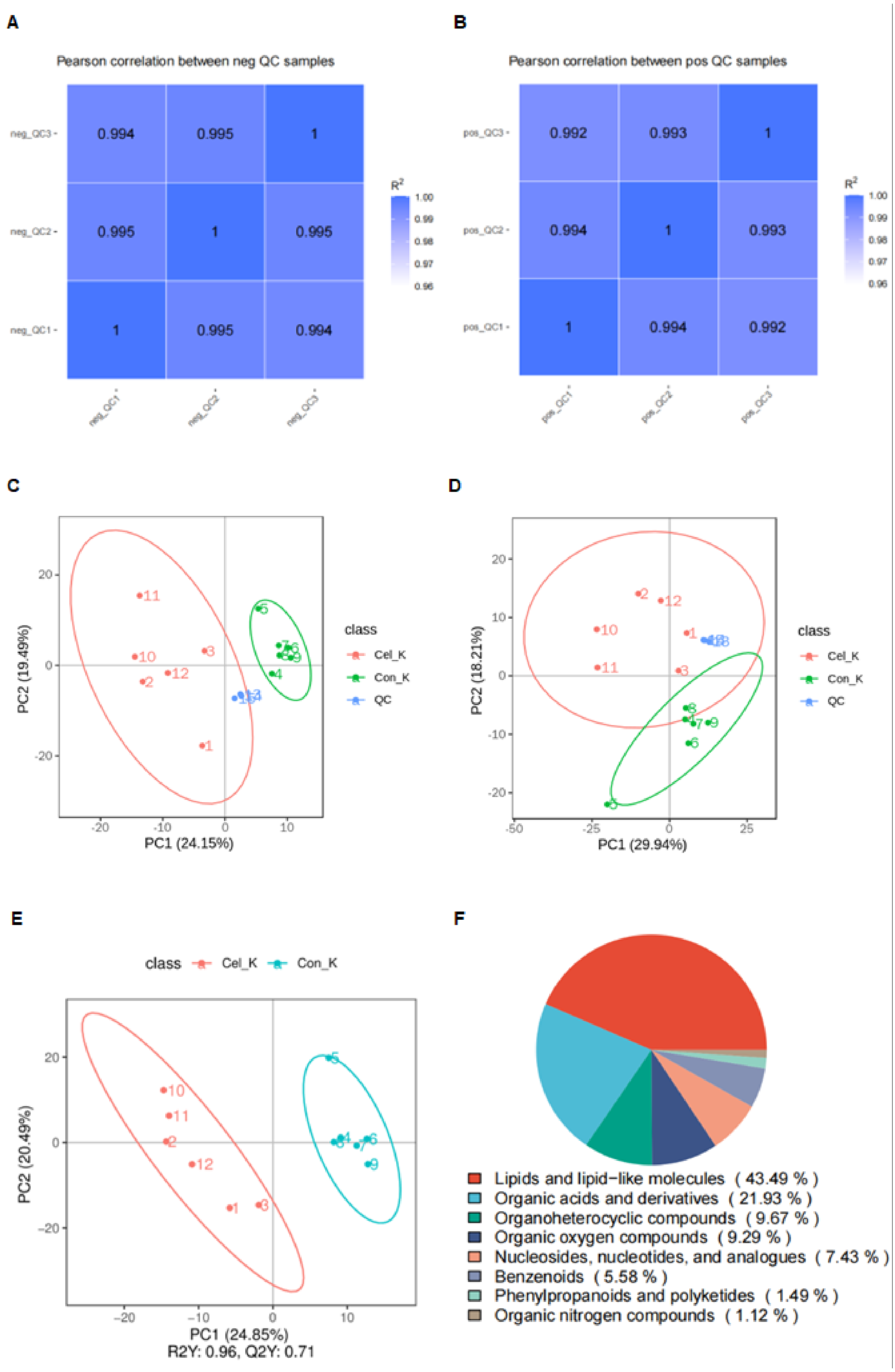

Supplementary Figure 6. Non-targeted metabolomics analysis demonstrated the metabolic differences after Cel treatment.

(**A** and **B**) Quality control (QC) of-kidney tissue samples in terms of the correlation in NIM and in PIM. QC PCA in NIM (**C**) and PIM (**D**). (**E**) Pos PLS-DA indicated an obvious difference between DMSO and Cel groups. (**F**) Pie charts showing the distribution of metabolites in NIM. Cel celastrol, QC quality control, PCA, principal components analysis, PC principal components, NIM negative ion mode, PIM positive ion mode, Pos PLS-DA positive partial least squares-discriminant analysis.
